# Flowering Mechanisms and Environmental Stimuli for Flower Transition: Bases for Production Scheduling in Greenhouse Floriculture

**DOI:** 10.3390/plants11030432

**Published:** 2022-02-05

**Authors:** Simona Proietti, Valentina Scariot, Stefania De Pascale, Roberta Paradiso

**Affiliations:** 1 Research Institute on Terrestrial Ecosystems (IRET), National Research Council of Italy (CNR), Porano, 05010 Terni, Italy; simona.proietti@cnr.it; 2 Department of Agricultural, Forest and Food Sciences, University of Turin, Grugliasco, 10095 Torino, Italy; valentina.scariot@unito.it; 3 Department of Agricultural Sciences, University of Naples Federico II, 80138 Napoli, Italy; depascal@unina.it

**Keywords:** floral induction, phase change, flower crops, ornamental crops, light intensity, light spectrum, photoperiod, temperature, vernalization, DIF

## Abstract

The scheduling of plant production is a critical aspect in modern floriculture since nowadays, sales are not oriented toward the recurring holidays as in the past, but always more toward impulse buying, implying a more diverse and constant demand on the market. This requires continuous production, often regulated by precise commercial agreements between growers and buyers, and between buyers and dealers, particularly in large-scale retail trade. In this scenario, diverse techniques to modulate the duration of the growing cycle, by hastening or slowing down plant growth and development, have been developed to match plant flowering to the market demand. Among the numerous approaches, the manipulation of climatic parameters in the growth environment is one of the most common in greenhouse floriculture. In this review, we summarize the physiological and biochemical bases underlying the main mechanisms of flowering, depending on the plant reaction to endogenous signals or environmental stimuli. In addition, the strategies based on the control of temperature (before or after planting) and light environment (as light intensity and spectrum, and the photoperiod) in the scheduling of flower and ornamental crop production are briefly described.

## 1. Introduction

The scheduling of plant production is a critical aspect in the horticultural industry, particularly in floriculture. Indeed, in this sector, a limited array of traditional products proposed to consumers in the last years have been gradually replaced by a broad range of items they had requested, for new and varying end uses [1]. In addition, nowadays, supplies are not mainly oriented toward the recurring holidays (e.g., Valentine’s day, Christmas) as in the past, but always more toward impulse buying, implying a more diverse and constant demand on the market. This requires continuous production, often regulated by precise commercial agreements between growers and buyers, and between buyers and dealers, particularly in large-scale retail trade [1]. In this scenario, diverse strategies to modulate the duration of the growing cycle, by hastening or slowing down plant growth and development, have been developed, particularly for greenhouse crops, in order to match plant flowering to the market demand.

In general, production scheduling in greenhouse horticulture allows for multiple advantages for the farm:

- optimizing manpower productivity and the efficient use of production facilities;
- allowing for the extension of the production period;
- increasing the commercial value of products by identifying convenient sale schedules;
- guaranteeing the constant availability of homogeneous products in terms of quantity and quality.

When products consist in cut flowers or flowering plants, production scheduling aims at controlling the flowering time and the aesthetical value of flowering. Among the multiple suitable approaches to regulate these features, the manipulation of climatic parameters in the growth environment is one of the most common and efficient tool.

This review summarizes the physiological and biochemical bases of the flowering mechanisms and the main strategies adopted in greenhouse floriculture for the production scheduling of cut flowers and flowering ornamentals. Particularly, we focused on the strategies based on climatic conditioning, including the control of temperature (before planting or during plant cultivation) and the light environment, in both quantity (intensity or duration) and quality (spectral composition). This approach aims at clarifying the scientific background of the most common practical interventions for forcing greenhouse crops in commercial floriculture.

## 2. Plant Growth and Development, Phase Changes

Plant production in modern greenhouse horticulture requires a fine control of plant growth and development throughout the entire crop cycle. While the terms “growth” and “development” are often confused in common language, they represent different plant processes; hence, it is important to distinguish between them as well as to identify internal and external influencing factors [2].

Plant growth is defined as the increase of plant mass, which can be measured on a weight basis (dry weight) or by other biometric parameters (e.g., stem height, leaf area) that describe plant size without using the destructive measurement of dry matter. Plant development consists of the anatomical and morphological changes occurring during a plant cycle and can be described as a sequence of successive phases over time, in qualitative rather than quantitative terms. Indeed, during their biological cycle, plants go through three distinctive developmental phases: the juvenile phase, the adult vegetative phase, and the adult reproductive phase [3]. The juvenile phase consists of an increase in plant size and mass, determined by the shoot and root growth, and is divided into several sub-phases: seed germination, the appearance of leaves and shoots primordia, and leaf and shoot growth. During the juvenile phase, the apical meristem continuously produces phytomers or stem primordia, including node and internode, with one or more leaves and axillary buds; the plant is unable to flower, even when exposed to optimal environmental conditions for flowering. The following phase is the adult vegetative phase, in which the meristem acquires the ability to flower (competence to flower). The last phase is the adult reproductive phase, which is characterized by the actual realization of reproductive structures after the occurrence of specific endogenous signals or environmental stimuli [4]. In fact, during this phase, specific internal and external signals promote substantial changes in the plant meristem: phytomers are replaced by flower primordia, and while the vegetative meristem is usually indeterminate, producing phytomers indefinitely, the floral meristem is determined, and the meristematic activity stops after flower formation.

The flowering process involves complex biochemical, anatomical, and morphological changes, synthetically described by four events: flower induction, flower evocation and initiation, and flower development. Flower induction consists in endogenous or exogenous signals determining changes in the plant developmental program. In response to these, a chemical stimulus is transmitted to the meristematic apex, which is altered to produce flowers instead of leaves in a process called floral evocation. This is followed by the formation of flower buds, defined as flower initiation, and by flower or inflorescence development [2].

**Figure 1:**
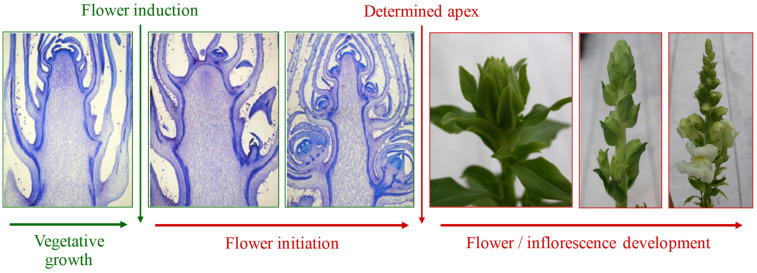
Subsequent phases of the development of the apical meristem and inflorescence in snapdragon (*Antirrhinum majus* L.). *Credit*: Roberta Paradiso.

The difference between the adult vegetative and reproductive phases consists of whether the meristem has been evoked to flower or is determined [2]. While at the macroscopic level, the beginning of flowering is identified with the appearance of the flower bud, the apex transition from one phase to another can be observed through microscope analysis (Figure 1).

## 3. Mechanisms of Flower Induction: Endogenous Signals and Environmental Stimuli

The transition of the shoot apical meristem from vegetative growth to flowering is the first step in the sexual reproduction of plants, and extensive studies combining cellular, molecular, and genetic approaches have elucidated several aspects related to this process and its control over time [5,6].

Flowering is a complex process, and still it has not completely been explored; however, it is now clear that it can be initiated in response to both endogenous signals and environmental stimuli [2,7]. Endogenous pathways are related exclusively to internal plant factors in a complex mechanism called autonomous induction, unaffected by environmental stimuli [3,8]. Plants with autonomous flowering react to a sophisticated network of endogenous signals, mainly depending on the plant age or size, the perception of seasonal climatic variation through the circadian rhythms, and the plant hormones [9]. For instance, plants can attain flower induction by reaching a certain age or a critical size of both the plant and the apical meristem. The relationship between plant size and the transition from the juvenile to the adult phase is linked to the number of leaves. In fact, it has been demonstrated that the plant may progress between the two phases only after the meristem grows beyond a minimum diameter, which grows as the number of leaves increases [2,10]. A certain leaf number (and therefore the related leaf area) is an index of the plant growth status required to produce an adequate quantity of floral stimulus in order to reach the competence to flower; it is also an indicator of metabolic reserves (i.e., carbohydrates) that support the high energy demand of flower and seed production [11]. The link between plant growth and competence to flower is confirmed by the evidence that environmental or cultural conditions promoting plant growth, such as high irradiance or nutrient availability, can reduce the duration of the juvenile phase, while unfavorable conditions which retard plant growth can delay flowering. For instance, reduced radiation and low temperature as well as water or nutrient deficiencies, which reduce plant growth, can prolong the juvenile phase or cause an extreme reversion from the adult to the juvenile phase [12].

Although autonomous induction represents an important mechanism of flowering, in most of the species, this occurs only after exposure to specific environmental conditions, particularly those affecting the photoperiod (duration of light and dark periods) and temperature [3,12]. In these species, flower induction requires a particular photophase or thermophase. The request for the inductive stimuli can be obligatory or qualitative when a certain condition is strictly required for flowering, or facultative or quantitative when the occurrence of this condition promotes (accelerates or enlarges) flowering, which is still possible in different conditions [13]. The control of temperature, photoperiod, and irradiance, and recently also of the light spectrum, is among the most common and efficient approaches to the control of flowering time used by scientists and modern horticulturists [13].

In relation to their photoperiodic requirement, plant species can be classified as long day plants (LDP)—induced to flower when the day length exceeds a certain threshold, short day plants (SDP)—which flower during a short day (or long night), and day-neutral (or indifferent) plants (NDP)—whose flowering is not dependent on the day’s length [2]. Two more groups of plants can be classified as long-short day plants (LSDPs), which require long days followed by short days for flowering induction, and short-long day plants (SLDPs), requiring short days followed by long days to flower [14]. Furthermore, some plants flower when the duration of light and dark is about 12 h each (intermediate day plants), while in others flowering occurs in short and long days, but not in intermediate days (ambi-photoperiodic plants) [2].

Several hypotheses have been suggested to explain how plants perceive the photoperiod, including the theory of a biological clock that works through circadian oscillations (lasting circa diem: about one day) of the day/night cycle [9,15]. It was demonstrated that plants recognize the photoperiod through mature leaves, which produce flower-triggering metabolites, translocated through the phloem up to the apex [16]. This theory was formalized as the florigen hypothesis by the Russian botanist Mikhail Chailakhyan (mentioned in [15]). To trigger the flowering response, the inductive photoperiodic conditions must persist for a minimum number of cycles, variable from one day to several weeks among the plant species; however, once the flowering stimulus is perceived, the apical meristem is determined, and plants can also flower in different environmental conditions.

The perception of the circadian clock and photoperiodism in photoinducible plants involves phytochromes and cryptochromes, photoreceptors of the red/far red and the blue wavelengths, respectively [17]. These photoreceptors act in the leaf by generating a complex network of light signal transduction, considered at the base of the photoperiodic control of flowering time in both SD and LD plants [18].

In addition to the photoperiod, light quality, and hormone biosynthesis and signaling, temperature represents an essential environmental cue that regulates the flowering transition. In fact, in some species, flower induction requires the exposure to low temperature until a certain cold requirement is fulfilled [5]. The length of the period of sensitivity to the thermal stimulus varies among plant species, and it can occur at a very early stage of plant development (i.e., seed) or can require the achievement of a certain size (i.e., number of leaves). The exposure to cold temperature to promote or accelerate flowering—and more generally, plant development—is known as vernalization, a phenomenon studied since the beginning of the last century [19]. Temperature is perceived in the entire plant organism, although low temperature is detected mainly by the shoot apex [5]. After vernalization, plants acquire the competence to flower, but in some species, this inductive effect can be delayed or reverted by de-vernalizing conditions such as high temperatures.

Light and temperature can interact in different temporal ranges, from a diurnal to an annual scale, so most of the plant species require a combination of a specific photoperiod and a certain thermal level in order to begin flowering [20]. For instance, in some species, flower induction from long days does not occur if a cold requirement is not previously fulfilled. This is because the same photoperiod can occur in different seasons (e.g., in autumn or spring); therefore, to reach reproductive success and survival, plants have developed sophisticated mechanisms to monitor environmental variables in order to flower only at the most favorable time.

In some cases, the flowering input depending on a certain condition (i.e., photoperiod) can be replaced by modifications of other factors (such as temperature or irradiance) [20]. In some LDPs (e.g., *Hibiscus moscheutos* L.), high irradiance can substitute the photoperiod requirement for flowering entirely [2]. Accordingly, in *Arabidopsis thaliana*, molecular studies have suggested that the integration of light and temperature signals in flowering regulation can involve the modification of the photoreceptor activity at different ambient temperatures [20].

Furthermore, light quality can have important effects on the flowering process [17]. In some NDPs (e.g., *Cyclamen* L.), light irradiance and/or spectral quality may modify the flowering time. More specifically, high irradiance and the blue (B) and red (R) wavelengths, when applied alone using LEDs, can anticipate flower bud induction and hasten the subsequent development [21]. However, plant response to light quality is species-dependent: in some LDPs grown under a high R:FR ratio (obtained by plastic filters absorbing FR from either sunlight or high-pressure sodium lamps) a delay in flower initiation (but not development) was observed in *Campanula carpatica* Jacq. and *Coreopsis grandiflora* Hoggs ex Sweet, and the inhibition of flower development (but not initiation) occurred in *Viola* × *wittrockiana* Gams. Conversely, in other crops such as *Lobelia* × *speciosa* Sweet and *Pisum sativum* L., an FR-deficient environment had no effect on flower initiation and development [22].

In many species, the interaction between the temperature and day length is the main factor for synchronizing flowering and the changing seasons, and is hence the main tool for the regulation of flowering time in greenhouse crop production. A good example of this is the SD plant *Chrysanthemum morifolium* Ramat, whose different cultivars can be classified into thermo-zero, thermo-positive, and thermo-negative based on their specific thermal needs. In thermo-zero and thermo-positive cultivars, used in year-round production, flowering proceeds rapidly at night temperature of 15–16 °C, with a slight inhibition below 10 °C for the first group and 15 °C for the second group; on the other hand, in the thermo-negative cultivars, flowering is inhibited at night temperatures higher than 15 °C during winter production. In the production scheduling of *Chrysanthemum*, year-round flowering is achieved through weekly transplants and a fine modulation of the photoperiod in heated greenhouses. In this respect, in SDPs, when the required long-night phase is interrupted by a short exposure to light (night break), the flowering process can be inhibited [23]. Hence, in *Chrysanthemum,* the night break can be effective in inhibiting flowering, particularly when red light is used, in order to allow for vegetative growth. This inhibitory effect can be reversed by the subsequent exposure to FR light, highlighting the involvement of phytochromes in the flowering response [23]. An opposite effect of the night break on flowering time was observed in LDPs grown under short-day condition, where a flowering acceleration could be induced, with a limited effect of the FR light exposure on this process reversion [24].

## 4. Control of Flowering Time in Greenhouse Floriculture

As flowering can be affected by multiple factors, including the intrinsic features of plant genotype, or the growth environment and cultivation conditions, diverse techniques in greenhouse floriculture that are based on genetic, physiological, and technological approaches have been developed to control flowering time. These techniques are able to modulate the duration of the growing cycle by hastening or slowing down plant growth and its developmental rate in order to match flowering to the market demand.

Strategies for production scheduling in greenhouse floriculture can involve one or both of the components of the plant–environment binomial. Accordingly, operations can be classified into three categories as they act: *i*) directly on the crop, *ii*) on the growth environment, or *iii*) on the whole production process through technologies involving the overall cultivation system (e.g., hydroponics).

In general, the duration of the different developmental phases within a plant species can vary with the plant genotype and is influenced by the environmental conditions, such that the choice of the variety, cultivar, or hybrid and of the planting date have an influence on flowering earliness as well as winter production, which will allow for the plants to reach the highest commercial value on the market. Flower crops typically scheduled with this strategy are herbaceous biennials or perennials, grown in annual cycles for cut-flower production, such as the snapdragon (*Antirrhinum majus* L.), prairie gentian (*Lisianthus russellianus* Hook), stock (*Matthiola incana* L.), and delphinium (*Delphinium* × *cultorum*).

In some perennial or biennial crops (snapdragon, lisianthus, stock), cultivars are chosen within groups of responses to climatic conditions in the different climates, and year-round cut-flower production can be achieved by alternating the different genotypes in the cold or heated greenhouses [25,26,27]. For instance, for the snapdragon, cut-flower production can be achieved all year round by selecting cultivars from four response groups recommended for cold greenhouses in the different seasons, with increasing requirement from the first to the fourth: from SD—low-light intensity and a night temperature of 7–10 °C, to LD—high radiation and a night thermal level higher than 16°C. Similarly, cultivars of the prairie gentian are classified into three groups suggested for transplanting in a heated greenhouse: first, from the end of October to the middle of February; second, from the end of February to the end of June; and third, from the middle of April to the end of June. As a consequence of the different earliness of genotypes and the improving climatic conditions, the time for flowering, from the first to the last, progressively decreases from about 110 days in the fall–winter cycles to 60 days in the spring–summer cycles. In stock, flower precocity depends on the duration of juvenility and is related to the number of leaves formed at the appearance of the first flower. Genotypes are grouped into extra-early cultivars, sown from April to May, with flowering in 10 weeks; early cultivars, sown from the middle of February to the end of March, with flowering in 14 weeks, or from December to January, with flowering in 18–16 weeks; late cultivars, sown from June to August, with flowering from October to March, in more than 18 weeks. Early types show a shorter juvenile phase (15 vs. 50 days) and develop a fewer number of leaves (10 vs. 60) compared to later types.

In remontant species (roses, carnation, gerbera), the time of transplanting is a relevant factor for modulating the distribution of plant production throughout the year; however, in these crops, further control of the flowering time and flowering characteristics (e.g., stem height and flower dimensions) can be achieved through other techniques such as pruning (e.g., roses), pinching (carnation), and thermal control (gerbera).

The choice of propagation material plays a crucial role in geophytes, as the size and the thermal history of the storage organ (bulb, tuber, rhizome, tuberous root) that are used for planting influence the time of development and characteristics of the flower stem [28]. Within the numerous species, crops can be classified on the basis of the time of flower induction and the period of flowering. More specifically, flower induction can occur in the propagation organ (generally called bulb) before the harvest (*Narcissus*, *Hippeastrum*), during storing (at the beginning, in tulip and hyacinth, or at the end, in crocus), or after planting, in the early (*Gladiolus*) or more advanced (*Lilium*) phases of cultivation. However, in some cases, the time of flower induction depends on the size of the bulbs. For instance, early flower induction in *Gladiolus* is possible in corms of large diameters, while in small corms, it is delayed to mid-summer or even autumn, when the effect of leaf number and area prevails over that of the season itself. Flower bulb crops can flower in spring (*Tulipa*, *Hyacinthus*, *Narcissus*, *Iris*), summer (lilies, calla lilies, *Gladiolus*), fall (*Amaryllis*, *Cyclamen hederifolium*), or winter (*Freesia*, *Hippeastrum*, *Crocus sativus*, *Ranunculus asiaticus*). In general, in geophytes with only one flowering, the production scheduling is based on the storing conditions of the bulbs (see paragraph 5.3).

In woody plants such as roses (*Rosa hybrida* L.) for cut flowers, flowering time is influenced by rootstock, thermal conditions, as well as the pruning and harvesting cut. This cut modifies the plant architecture, influences the plant metabolism, and promotes the emission of flower stems by releasing the apical dominance, while temperature is the main factor affecting the growth rate after the bud breaking [29]. Pruning can be performed periodically or can coincide with the harvest. The height of the cut depends on the plant vigor: in general, a lower cut will leave the plant with a few buds, which will form fewer but more vigorous stems, while a higher cut will leave more buds, which will give more stems with lower-quality characteristics (stem length, number of leaves, blossom size). The time for flowering varies from 6 weeks in spring and fall to 10 weeks in winter, depending on the cultivar, the kind of cutting, and the cultural and environmental conditions. Temperature control implies knowledge of the thermal requirement of each variety, which allows for the exact prediction and scheduling of the harvest.

Within the cultural practices, the application of growth regulators allows for the modulation of the rate of the different developmental phases through chemical reactions in numerous plant species [30]. Some plant hormones such as gibberellins, jasmonic acid, abscisic acid, and auxins have important effects on flowering [31]. In particular, treatments with exogenous gibberellins can replace the photoperiodic inductive signal in some photosensitive plants, mainly LDPs. Products containing gibberellic acid (GA3), applied as foliar spray, can be used during cultivation to promote flowering in LD biennial species of rosette *habitus* (e.g., *Calendula*, *Dianthus*), in which GA3 anticipates flower induction and promotes floral and inflorescence development. This effect can also be obtained in short day conditions and without the cold exposure [32].

The application of GA3 through rhizome submersion or foliar spray promotes flowering in potted ornamentals of the Araceae family (e.g., *Anthurium*, *Spathiphyllum*, *Zantedeschia*) [33]. Some extremely active chemicals such as paclobutrazol, chlormequat chloride, and daminozide (chemical family triazoles) are used as growth regulators, affecting flowering in almost all plant species, whether applied as a spray or a soil drench [34]. The growth-regulating properties of these compounds are mediated by the inhibition of gibberellic acid biosynthesis and by the increase of cytokinins and abscisic acid levels. Depending on the plant species and the timing of application, chemical growth regulators can delay or promote flowering. Paclobutrazol and daminozide retard the flowering of chrysanthemum, to an extent dependent on the product concentration, and may delay the flowering of bedding plants. Conversely, chlormequat chloride promotes earlier flowering and increases the number of flowers in *Hibiscus* L. and geranium (*Pelargonium graveolens* L.) [35]. Daminozide also affects flowering in some woody ornamental plants, such as *Rhododendron* [36], and delays inflorescence development in azalea [37]. Historically, triazoles have produced the best compounds for ensuring suitable growth control in a wide range of crops [38]. More than the compounds mentioned above, other triazoles are used successfully on flowering crops such as flurprimidol, known for its effect on woody ornamentals, including the acidophilic species *Rhododendron catawbiense* (Michx.). In *Camellia japonica* L., flurprimidol increases the number of flowers and is an effective alternative to paclobutrazol, which is less harmful to the environment [39].

Ethylene is the only natural plant growth hormone, which exists in gaseous form and is effective in inducing flower development [35]. However, the role of ethylene in floral induction is complex and controversial since plant species can show opposite responses due to the interaction of ethylene with other hormones during flower development [40]. On the other hand, exogenous ethylene (gaseous ethylene or ethylene-releasing substances such as ethrel) has been widely used to induce the flowering of bromeliads (*Guzmania*, *Aechmea*, *Neoregelia*, *Tillandsia*, *Vriesea*), in which it synchronizes and accelerates flowering [40,41]. Conversely, some ethylene synthesis inhibitors such as silverthiosulfate, amino-oxyacetic acid, and aminoethoxyvinylglycine (AVG), which are also very active at low concentrations, are known to interfere with flower induction, resulting in a significant delay of flowering in Bromeliaceae [41].

In flowering potted plants, controlling plant size and shape in addition to the time of flowering is also important. For this purpose, the application of growth retardants allows for the limiting of stem elongation in order to obtain more compact plants, while improving foliage greenness and flowering [42]. In addition, cytokinins and ethylene-releasing compounds (dikegulac sodium and methyl esters) promote the development of shoots of lateral branches through the release of the apical dominance and the break of dormant buds, and hence the formation of denser foliage.

## 5. The Role of Temperature in Flowering

Growth temperature is the factor most frequently controlled in the greenhouse, directly through heating and cooling, or indirectly through ventilation and humidification. The control of temperature, both upwards and downwards (due to the high levels reached in the Mediterranean climate), allows for the regulation of the plant biological clock by acting on the thermal time. The literature discusses three types of temperature: air, leaf, and substrate. Generally, air temperature is the most considered, as it is the easiest to monitor and control; however, the actual leaf temperature can differ greatly from the air temperature, depending on the relative humidity and the consequent transpiration rate. Conversely, the substrate temperature reflects the actual root temperature.

In general, the speed of enzymatic processes and the growth rate increase with increasing temperature, reducing the duration of each phase and the transition time to the next phase. These effects, however, occur within specific ranges: i) the base temperature (T_base_), below which the plant stops growing and developing (while remaining vital); ii) the optimal temperature (T_opt_), at which growth and development are achieved in a balanced way at maximum speed; iii) the maximum or critical temperature (T_crit_), beyond which the growth stops. Each plant species has a range of optimum growing temperatures and a range of tolerable temperatures. The former produces high-quality plants most rapidly, while the second may result in long production time and low product quality. Knowledge of the values of these temperatures, called cardinal temperatures, is fundamental for the air conditioning of greenhouses (Table 1).

On the basis of the T_base_, plant species are classified into microtherm (2–6 °C), mesotherm (6–10 °C), and macrotherm (10–14 °C) species. The prevalence of ornamental and cut-flower crops belongs to the mesotherm (e.g., rose, gerbera) or macrotherm (main orchids) groups; hence, in many of them (e.g., rose, gerbera, and tropical orchids), temperature control is necessary to succeed in greenhouse cultivation and represents the main tool for regulating the flowering time (Table 1).

The species-specific T_min_ or T_opt_ depend on the natural origin and distribution of a particular species. Additionally, breeding has introduced desirable traits such as early flowering as well as frost and heat tolerance. Therefore, depending on the breeding objective, cultivars of the same species may have different T_min_ and T_opt_ values as well as varying thermal time for a particular event such as flowering. For example, the calculated T_min_ for petunia (*Petunia* × *hybrida* Vilm.-Andr.) ‘Dreams Neon Rose‘ is 2.7 °C lower than that for ‘Wave Purple Classic‘ [43]. The T_min_ and T_opt_ values within a species can also vary with the developmental or physiological process, and environmental conditions such as the photoperiod and the daily light integral (DLI) [44]. The estimation of T_min_ and T_opt_ allows growers to categorize crops according to their temperature sensitivity and to manage homogeneous groups for an energetically efficient production.

The time to reach a particular developmental event as a function of temperature can be expressed as the number of days required to reach that event (n) or as a developmental rate, calculated by taking the reciprocal of that number of days (1/n). Temperature influences flowering time, such that as mean daily temperature (MDT) increases within a species-specific range, days to flower (DTF) decreases. When plants are grown at an MDT above the optimum temperature (T_opt_), the flower development rate begins to decline [43]. This phenomenon is known as *heat delay* and may be due to a delay in flower induction, initiation, and/or development. In many ornamental plants (e.g., *Viola* and *Catharanthus roseus* L.), a linear function can be used to describe the relationship between the flowering rate and the MDT when the MDT is between the T_min_ and T_opt_. However, in some crops, a quadratic function better describes the effect of the MDT on the DTF, as a decrease in the DTF with an increase in the MDT is not linear. For example, there can be a greater decrease in flowering time when the MDT increases from 15 to 20 °C than when it increases from 20 to 25 °C [44].

The flowering response to the MDT varies widely among species and cultivars, and it has been quantified for some annual crops (e.g., bedding plants). The authors of [44] determined how the MDT controlled the flowering time of 18 cultivars of 16 ornamental species of herbaceous annuals (such as *Antirrhinum*, *Calendula*, *Impatiens*, *Matthiola*, *Petunia*, and *Tagetes*). As the MDT increased from 14 to 26 °C, the DTF decreased for all crops, except *Impatiens walleriana* Hook. Based on the results, mathematical models were generated to predict thermal flowering rates to improve production scheduling in the greenhouse.

The effect of temperature can be reflected in the entire growth cycle, in single phenological phases (e.g., from sowing to flowering), or in specific organs (e.g., fruit formation), defining a thermal time for each. According to this model, the duration of the cycle or of a single phase depends on the “heat” accumulated by the plant, defined as ΣGDDi, the sum of the GDDi (growing degrees day), calculated on a daily basis as the difference between the mean air temperature (T_mean_, as the mean of the minimum temperature T_min_ and the maximum temperature T_max_) and the T_base_, and referred to the i-th day: GDDi = (T_mean_ − T_base_)i. This approach implies that the days during which the temperature is below or above the limits for the species (T_base_ and T_crit_, respectively) do not contribute to the calculation as they are not effective for growth.

In some flower and ornamental crops, the critical dimension for flowering occurs when a thermal sum (indicating the satisfaction of the specific temperature requirement for the cultivars) is reached. The time (X)/temperature (Y) relationship is of the hyperbolic type (X*Y = constant) and indicates the possibility of programming production by increasing the ambient temperature, with a consequent reduction in the duration of the phases. However, it should be considered that, similar to all models, the thermal age model is based on some simplifications: the relationship between growth and GDD is linear (which is not always true); the development, even in the initial stages (germination, emergence, formation of the first true leaves), depends on the temperature of the air more than that of the substrate; the effects of thermal excursion are negligible; the culture is in ideal conditions (e.g., water and nutrient supply) and never exposed to abiotic or biotic stress. Despite these approximations, the model is useful in programming different species for which the cardinal temperatures and the plant response are known in environmental and cultural conditions similar to those of interest. For instance, the thermal sum calculated from transplanting (or pruning, in the case of the rose) to flowering ranges around 400–600 GDD in tulip, 500–600 GDD in iris, 700–800 GDD in rose, and 1000–1400 GDD in chrysanthemum. Obviously, for heating to be effective in promoting development and anticipating flowering, optimal levels must be guaranteed for all the parameters, even with other climate interventions (e.g., artificial lighting, carbon fertilization), and growth has to be supported by adequate inputs (e.g., water and mineral nutrition).

### 5.1. Day-Night Differential

In most plants, the night temperature has a greater effect than the day temperature in determining some physiological responses such as flowering (Table 2).

For example, high night temperature may cause a delay in flower initiation in *Chrysanthemum*, *Poinsettia*, and *Kalanchoe* and bolting in *Petunia*, while low night temperature may induce premature flower initiation or dormancy [45]. However, for many species, it is still unclear if day, night, or average daily temperatures are the most efficient in obtaining a specific plant response. For instance, *Euphorbia pulcherrima* Willd. is more sensitive to the night temperature, while *Antirrhinum* reacts more to the day temperature, and *Pelargonium* spp. is more responsive to the average daily temperature [46].

In many cases, the higher the day temperature relative to the night temperature (day–night = DIF), the greater the stem elongation; conversely, a cool temperature for at least two hours just before the first light in the morning (negative DIF) reduces stem elongation. This early morning cool pulse is known as the temperature drop. The effects of the DIF and the temperature drop on plant morphogenesis for a wide range of plant species were summarized by [46]. Although they affect stem elongation and plant height more, the DIF and temperature drop may influence the number and size of the flowers in some species. In LDPs such as *Campanula isophylla* Moretti and *Fuchsia* × *hybrida* L., the DIF only had a minor influence on the time for flower initiation and the rate of flower development.

The effect of negative the DIF on flowering is different for the SDP poinsettia, as flowering is delayed significantly. The effect of the temperature drop on flowering is rather small on SDPs such as *Euphorbia pulcherrima* (Willd.) and *Begonia* × *hiemalis* Fotsch.

The DIF and temperature drop interact with growth factors such as photoperiod, irradiance, light quality, and growth retardants, probably affecting the concentration of endogenous gibberellins (GAs) [46]. As an example, in the geophyte *Sandersonia aurantiaca* Hook., the DIF significantly affected flower stem parameters (length, strength, and flower numbers), but a high irradiance is required to avoid a reduction of the stem strength [47].

### 5.2. Vernalization

In temperate climate, many annual, biennial, and perennial plants perceive cold as an environmental signal to flower at the proper time of year. More specifically, certain species need to experience a period of low temperature to overcome a block to flowering; this occurs through a process known as vernalization. This is an adaptation strategy, implying complex physiological and metabolic changes that prevent flowering before winter while promoting it under the favorable conditions of spring [48].

Molecular analyses revealed that vernalization typically represses the expression of floral repressors that block flowering before the winter cold exposure, thus enabling plants to become competent to flower in spring or early summer in response to increasing day length (i.e., LD signals) [49]. Chen (2020) [50] revealed a regulation network for the early development of flower buds under low temperature induction in *Phalaenopsis amabilis* L., one of the most important orchids in the international flower market, which requires low temperatures for flowering.

The cold requirement can be obligate (cold is necessary for flowering) or facultative (cold hastens flowering). In both cases, vernalization is a quantitative process, and the flowering response increases with the exposure to low temperature until “saturation” [51]. Conversely, insufficient vernalization can result in incomplete or delayed flowering, which can cause considerable monetary losses to growers. In general, after the fulfilment of vernalization requirements, exposure to higher temperature is needed for the subsequent flowering [52].

Vernalization has been studied in many winter annuals and biennials and selected perennials. Many vernalization-requiring crops are LD plants. The effective temperature and the length of cold exposure that are required can vary among the plant species and varieties [48]. For example, the temperatures producing the greatest flowering response in *Veronica spicata* L. ‘Red Fox’ and *Laurentia axillaris* (Lindl.) E. Wimm. were −2.5 °C and 5–10 °C, respectively [53], while the complete flowering of *Campanula* ‘Birch Hybrid’ was achieved after a five-week vernalization treatment at 2.5–7.5 °C or after a nine-week treatment at 0–12.5 °C [52]. Therefore, the selection of appropriate inductive temperatures is important for commercial production of herbaceous perennials. Even within a species, the optimum vernalization temperature range may broaden with increasing duration [51].

In some species, the cold treatment can be applied to seeds or storage organs (bulbs, tubers, corms, or rhizomes), and a certain moisture level is often required for the cold to be perceived, while in others, only mature plants are responsive. The duration can vary widely with the species, from a few days to several weeks, and the temperature can vary from just above 0°C to much higher values.

The quantity of cold treatment needed for the resumption of growth is defined as the chilling requirement. The amount of chilling units (CU) may be calculated according to the Utah Chill Unit model [54]. In this model, 1 h at temperatures ranging from 2.5 to 9°C provides 1 CU, and 1 h at 9–12°C provides 0.5 CU. Conversely, 1 h at a temperature between 16 and 18.5 °C decreases the amount of accumulated CU by 0.5 units, while 1 h at more than 18.5 °C decreases the accumulated CU by 1 unit.

The role of low temperature in the plant’s life cycle varies with the species, and three categories can be distinguished [45]:

(i.) Species requiring cold for growth and development (endodormancy). For example, a cold treatment is required by *Tulipa* for shoot elongation and flowering, by *Lilium longiflorum* (L.) for flower induction and initiation, by azalea (*Rhododendron* L.) for the development and anthesis of flowers previously formed. In *L. longiflorum* ‘White Heaven’ bulb exposure to 4 °C for one week induced a decrease of about 20% in the time needed for floral transition [55]. Additional cold exposure led to a gradual decrease of about 80% and 55% for floral transition and flowering, respectively, after nine weeks at 4 °C;
(ii.) Species requiring cold for continued growth and development after an induced dormancy, but the plant can complete its life cycle and flower without a dormancy period. For example, tuberous begonias grown under a 12-h day length or less develop dormancy and need exposure to a cold period in order to break it and to flower;
(iii.) Species for which cold is not required but prevents growth and development and reduces desiccation (ecodormancy). For example, *Hippeastrum* bulbs stored at 5–9 °C delay flower and leaf emergence, allowing storage and shipping.

Once completed, the vernalization process can be very stable, and in some species, SD, LD, or GA may partially or totally replace the cold treatment. For instance, *L. longiflorum* bulbs exposed to vernalizing temperatures retained that information even when several warm periods occurred prior to the completion of vernalization. LD may substitute vernalization in part (after 1–2 weeks of cold) in Eastern lilies; GA could completely substitute the cold treatment in azalea after flower buds have developed [45].

### 5.3. The Control of Temperature for Scheduling Geophytes

Ornamental geophytes include more than 800 different genera, 60 of which are used for flowering potted plants, cut flowers, and garden ornamentals, although industrial production is dominated by seven genera: *Tulipa*, *Lilium*, *Narcissus*, *Gladiolus*, *Hyacinthus*, *Crocus,* and *Iris*.

Geophytes are particularly suited to face unfavorable climatic periods. According to their origin, the two main climatic cycles to which these organs are adapted are the cold–hot cycle of temperate zones and the humid–dry cycle of tropical and subtropical areas, including the Mediterranean region. Being native of climatically difficult environments with a long adverse season has meant that these plants require the timely fulfilment of certain thermal and light needs; hence, in intensive floriculture, they are frequently subjected to forcing in order to plan flowering to match the recurrence market.

In most crops and in natural conditions, leaves wilt in the summer, and a period of dormancy starts until their exposure to winter cold temperature. Based on this cold requirement, bulbs are vernalized in commercial practice, with thermal levels and time of exposure varying according to the plant species [56].

Several flowering geophytes (*Tulipa*, *Iris*, *Lilium*, *Gladiolus*, *Ranunculus*, *Dahlia*, *Narcissus*) are scheduled through the choice of the preparation procedure for the propagation material, in which the cold requirement can be partially or totally fulfilled. For instance, in tulip (*Tulipa* spp.), cultivation in the Mediterranean or temperate climate can be carried out in an open field, and flowering occurs from March to May; cultivation can also be performed in the greenhouse, with flower production all-year-round, for both non-treated and vernalized bulbs [57,58]. During the storing, the bulb growth continues until the development of the flower primordia of the gynaecium (Stage G). The numerous commercial hybrids, with a broad range of shapes and colors, are classified in early flowering, with medium flowering earliness and late flowering, and the procedure of cooling varies with the genotype and the production schedule. More specifically, two approaches can be adopted, with the cold requirement fulfilled completely before planting (5 °C tulips) or during the bulb storing and after planting (9°C tulips) [59].

Based on the different dormancy traits, the major geophytes can be classified into three groups: (1) species with a relatively long dormant period, during which the differentiation of new organs is inhibited (e.g., *Gladiolus*); (2) species that differentiate flower buds inside the bulb before/during dormancy (*Tulipa*, Asiatic *Lilium* hybrids, and *Hyacinthus*); (3) species with no visible dormancy unless in harsh environments (e.g., *Hippeastrum*).

Temperature is the main factor in geophyte dormancy, affecting internal metabolites, phytohormones, and signal response [60]. Most species do not show preference for a particular photoperiod, although in a few geophytes that respond to both temperature and day length (e.g., *Colchicum tunicatum* Feinbr.), flowering is generally facilitated by short days [61].

Basic protocols for breaking the dormancy of selected ornamental geophytes were reviewed by Dole (2003) [62]. Most geophytes require a “warm–cold–warm” sequence to complete their life cycle. Different genera and species demand various temperature optima, but in general, the optimum temperature for flower meristem induction and the early stages of floral organogenesis normally ranges from 15 to 21 °C, while the temperature for final flower formation ranges from 4 to 9 °C [63]. The ecological origin of the species delineates the physiological requirements: for example, species from temperate zones (e.g., *Allium* and *Iris*) usually require low temperatures for optimal flower differentiation (9–13 °C), species with thermoperiodic cycle that originate from the Irano-Turanian regions (e.g., *Tulipa* and *Hyacinthus*) require relatively high temperatures for differentiation (17–25 °C) and a period of lower temperatures (4–9 °C) to allow floral stem elongation and anthesis; on the other hand, autumn-flowering geophytes of the Mediterranean region (*Crocus*) are adapted to warm environmental conditions [64].

Numerous studies have shown that the effects of the temperature surrounding the underground organs during the autumn–winter period can lead to important physiological changes in plants. Khodorova and Boitel-Conti (2013) [61] reviewed experimental data on temperature requirements for flower initiation and development, shoot elongation, aboveground growth, and anthesis in bulbous plants. In addition, they examined the physiological processes occurring during the autumn–winter period in bulbs (water status, hormonal balance, respiration, carbohydrate mobilization) and how these changes might provoke disorders in stem elongation and flowering.

### 5.4. The control of Temperature for Scheduling Ornamental Shrubs

Ornamental shrubs of temperate zones such as *Camellia* L., *Rhododendron L.* and *Hydrangea* L. require flowering to be abundant, early, and synchronized in order to reach high marketable quality. Flower initiation and the early differentiation of flower primordia start in late spring, while flower bud development and visible bud enlargement continue until autumn. At this point, as with the majority of temperate woody plants, they exhibit a well-defined dormancy or resting phase.

For most species, bud dormancy or endodormancy is induced by a decreasing photoperiod, while the release is generally stimulated by extended periods at low temperature. This is the case of genera such as *Rhododendron* [65] and *Hydrangea* [66], where the exposure of floral buds to cold (2–7 °C) stimulates endodormancy release and promotes normal growth and anthesis during the following spring. However, warm spells during late autumn and winter can lead to an unfulfilled chilling requirement, leading to erratic and delayed flowering. A standard practice for *Rhododendron simsii* is the storage plants with fully differentiated and developed endodormant flower buds in the dark, at low temperatures, for up to 8 weeks [65]. In *Camellia japonica* ‘Nuccio’s Pearl’, storing plants at 7 °C for 4, 6, and 8 weeks of cold provided 672, 1008, and 1344 CU, respectively [67]. The results indicated that 6–8 weeks of cold storage promoted dormancy breaking, leading to an increased percentage of flowers that successfully reached the anthesis, a lower need for forcing, and a more uniform flowering. Similarly, in *Hydrangea* L., *Paeonia* L., and *Helleborus* L., the required days for forcing depended on the extent of the cold treatment [67]. However, in camellia, the cold treatments also showed some drawbacks in flower quality, such as a reduction in flower size, anthocyanin content, and flower longevity, probably due to the dark conditions. Thus, for some species, attention should be paid to overcome eventual quality-related problems, e.g., by carrying out the cold treatment at an earlier bud developmental stage to avoid interference with floral pigmentation, and by applying a photoperiod and slightly higher dormancy-breaking temperatures to stimulate photosynthesis.

### 5.5. The Control of Temperature for Scheduling Orchids

The Orchidaceae family has over 880 genera and 25,000 species distributed around the world. Many orchid genera are induced to flower by low temperature: *Dendrobium* Sw. (10−13 °C), *Cattleya* Lindl. (12−16 °C), *Mitoniopsis* (11−14 °C), and *Zygopetalum* Hook. (11−14 °C) [68]. *Phalaenopsis* also requires a temperature reduction for the transition from the vegetative to reproductive stages. More specifically, vegetative growth is maintained above 28 °C, while flowering initiation and inflorescence development requires 17–25 °C and 17–26 °C, respectively. Thus, controlling temperature to schedule flowering is crucial for the year-round production of this orchid. In practice, commercial growers can maintain vegetative growth during the cool season (<25 °C) by heating the greenhouse to above 28 °C, and flower induction can be stimulated during the warm season (>28 °C) by cooling it down to below 25 °C [69].

Due to the high thermal requirement, greenhouse heating is one of the main expenses for *Phalaenopsis* production in Mediterranean areas; hence, the technical adjustment of inductive temperatures and treatment duration, based on the hybrid-specific sensitivity, could contribute to reducing the operating costs and improving the economic benefits [70].

## 6. Light Intensity, Photoperiod, and Light Spectrum

Plants use light both as an energy source for carbon fixation in photosynthesis (assimilative function) and as a signal to activate and regulate many other key processes of plant growth and development (control function) [17].

In modern horticulture, several technologies are applied to improve the light environment in the greenhouse for both plant functions, including innovative cover materials and artificial lighting. For instance, diffusive and photoselective films are respectively used to increase the light transmission and the use efficiency of solar light in the greenhouse, or to modify its spectral composition to drive plant metabolism toward specific responses (e.g., biosynthesis of metabolites such as antioxidants or pigments) [71]. On the other hand, assimilative lighting allows for the maximization of photosynthetic performance in both vegetable and flower or ornamental crops, mainly in the presence of unfavorable outdoor conditions or high cultivation density, to increase plant productivity and to achieve constant yield and product quality [72].

In plant species with a high requirement for light intensity, supplemental lighting is applied to integrate with the natural light when solar radiation is insufficient or variable during the day (e.g., in regions of Northern Europe, during the winter season). For this assimilative function, the intensity of artificial light is commonly at 100–200 µmol m^2^s^−1^ (useful for photosynthesis), and the duration of the lighting period per day depends on the desired response. High-intensity discharge (HID) sources such as metal halide (MH) and high-pressure sodium (HPS) lamps are typically used in greenhouse horticulture for assimilative lighting; however, the use of light emitting diodes (LEDs) is increasingly more extensive [17]. LEDs offer several advantages compared to the conventional sources, such as the possibility to regulate the light intensity and to tailor the light spectrum, depending on the specific requirements of the different crops and development stages [73]. As a consequence, alone or in addition to traditional lamps, they allow for photosynthetic assimilation as well as the control of plant photomorphogenesis and metabolism [74].

In Northern Europe, important flower crops (i.e., rose, gerbera, and lilium) are lighted through lighting programs, providing a minimum daily threshold of radiation or a specific daily light integral (DLI) at the base of species requirements [75]. Conversely, in some Mediterranean agricultural areas, light environment in the floricultural system remains largely uncontrolled, and the seasonal trend of solar radiation impacts on the production scheduling [76].

In plant species responding to variations in the photoperiod, control of flowering can be achieved by modifying day length through the application of black screens or artificial lighting. These procedures are widely applied in both SDP and LDP, exhibiting an obligate response (flower induction occurs only when the dark period is respectively longer or shorter than a critical duration) or a facultative response (a certain photoperiod induces a faster and more abundant flowering, which would still occur under a different photoperiod). The dark or light duration inducing flowering is called critical day length (CDL). Each species is characterized by a specific CDL, but this has a different meaning in SD and LD plants. In fact, to induce flowering, the photoperiod must be shorter than the CDL in SD plants and longer in LD plants. For instance, chrysanthemum and tuberous begonia have similar CDL (about 13 h), but chrysanthemum is an SDP, while begonia is an LDP. When the CDL consists in the duration of dark time, the conditioning procedure involves the use of black cloths, usually from the afternoon until sunset, in order to extend the dark period. On the contrary, when the CDL represents the duration of the light phase, this can be extended by using artificial lighting, applied before or after the natural light period (day extension) or as a night interruption (night break).

Among obligate SDPs, typical examples are chrysanthemum (*Dendranthema* × *morifolium* (Ramat.), with a CDL of ≥13.5 h for vegetative growth and a CDL ≤ 12 h for reproduction), poinsettia (*Euphorbia pulcherrima* Willd., CDL 12.5 h), *Kalanchoe blossfeldiana*, (Poelln.), some orchids of *Cattleya* genus, and some begonia species. *Chrysanthemum* is a highly scheduled crop, with a very specialized cultivation technique. Greenhouse (cut flowers and flowering plants) and garden (cushion and upright types) chrysanthemums provide a huge diversity of flower forms and colors and plant habits. Cultivars and hybrids are classified into groups of early, mid, and late response, depending on the number of weeks of SD required for the flower bud initiation (beginning of inductive light conditions) and the complete inflorescence development, which is called the time of reaction (TR) [77]. In early flowering genotypes (TR 6–8 weeks), the requirement for SD is facultative for flower bud initiation but obligate for flower development, while late flowering types (> 8 weeks) have obligate SDP for both phases [2]. A close interaction between photoperiod and temperature influences the flowering of chrysanthemum in late summer and fall; hence, year-round production involves the manipulation of both parameters [78]. When plants are exposed to a photoperiod exceeding 13.5 h after flower bud initiation, bud development is interrupted [2,79,80]. The year-round production also requires weekly staggered transplanting and photoperiodic reduction with shade nets, as well as an extension with artificial lighting in a heated greenhouse. In autumn and winter, when SD induces flowering, plants are kept in vegetative growth for the first 3–5 weeks after planting through night break lighting until an adequate plant size is reached; subsequently, the end of lighting and the exposure to SD promotes flower induction. The duration of darkening depends on the TR, which varies from 6 to 15 weeks, depending on the variety (usually 8–11 weeks). In most varieties, the flower primordia are visible in 3–4 weeks, and darkening is applied until color is visible. The crop scheduling involves the division of the greenhouse into a number N of sectors, where N is the number of weeks from transplanting to harvest, in which plants are transplanted every week, and flowers are harvested. On the contrary, in spring and summer, after the growth period, the short day required to trigger flowering is obtained by black screens.

In facultative SD species, flower development is accelerated by a short photoperiod, which is not essential for flowering. This is the case for some pot flower species such as *Begonia elatior*, *Tagetes erecta* L., and *Tagetes patula* L., which are cultivated for a dual purpose (pot flower plants and cut flowers). However, in some geophytes (e.g., *Gladiolus* spp.), the photoperiod interacts with temperature, influencing the allocation of photosynthetic products and the growth of the hypogeal or epigeal part of plants, thus influencing earliness and flower quality. It should be emphasized that in SD crops, darkening and artificial lighting promote and delay the flowering processes, respectively, showing how the careful control of the photoperiod represents an additional and remarkable scheduling tool.

In obligate LD plants, flower induction occurs only when the photoperiod is above a certain threshold. The FC varies according to the species: it is 14 h for many begonias (*Begonia* spp.), 12 h for *Fuchsia*, 14 h for some species of *Rudbeckia* L. In LD plants, photoperiodic light is applied to simulate a day longer than the natural photoperiod, and because it can be perceived at very low intensities, it is supplied at levels of 1–3 µmol m^−2^ s^−1^, for a daily variable duration according to the cultivation period and the needs of the species.

In facultative LD species, flowering is accelerated and more abundant during a long photoperiod, even though it can occur under SD. This is the case for widespread crops such as snapdragon, *Lisianthus*, carnation (with great variability between cultivars), *Petunia*, *Gladiolus* spp., *Alstroemeria hybrid*. These were originally LDPs, with some selections also able to flower under SD. Moreover, in LDP, the CDL can be influenced by temperature (especially during the night) and sensitivity to the thermo-photoperiod and to light intensity, which varies for every cultivar, depending on the geographic origin of parental varieties from which the hybrids are obtained.

In some flower crops, production scheduling requires the combination of multiple approaches. This is the case of bedding plants, flowering plants for flower beds, and outdoors spaces, grown mainly from seeds in short cycles. Despite bedding plants representing a very heterogeneous group in terms of botanical classification and biological cycle (annual, biennial, and perennial), these plants are grown with a similar cultivation technique [81]. In general, they are classified according to the length of cultivation cycle, from sowing to flowering, variable from 4 to 16 weeks. The forcing technique consists in controlling the substrate temperature and the light or dark conditions (obtained by covering seeds with the substrate) during germination, and in the assimilative and photoperiodic lighting during the growth in the nursery (Table 3). 

Four growth phases have been defined: from the sowing to the emission of roots (I), from this to cotyledon expansion (II), from this to the appearance of all the true leaves (III), and to the final plant size and flowering (IV or finishing). The accurate prediction of production time is achieved through strictly controlled conditions, in different sectors of the nursery, in which species with similar requirements are kept under the same temperature and light irradiance and duration. An important practice for the scheduling of bedding plants is also the application of growth retardants, in order to reduce stems elongation, to obtain compact plants, and to promote flowering [82].

## 7. Conclusions

The scheduling of plant production is a crucial aspect in the modern floricultural industry, as the market demand for a continuous supply of very diverse items imposes the constant production of a multitude of plant species, with a wide range of cultural and environmental requirements.

The increasing knowledge in plant physiology, photomorphogenesis, and metabolism as well as the advances in greenhouse technologies through time have paved the way for the development of multiple strategies in order to finely regulate the rate of plant growth and development in numerous crops.

At present, most of the flower crops can be precisely scheduled, thanks to specific approaches involving one or both of the components of the plant–environment binomial, hence implying operations directly on the plant, on the growth environment, or on the whole production process.

In many crops, year-round production is achieved by controlling the flowering time with the combination of several approaches, often including the manipulation of temperature (before or after planting) and the light environment (as light intensity and spectrum, and the photoperiod). This control also relies on the availability of genotypes that are well-characterized for their response to environmental stimuli.

Though research on both the crops and the greenhouse technologies for climatic control have been making fast progress in the last years, several research gaps still need to be solved in order to achieve efficient and economically and environmentally sustainable production in floriculture. For instance, studies are warranted on the precise thermal requirements in some vernalizing species (e.g., emerging geophytes), on possible strategies alternative to heating in high temperature-requiring plants (e.g., tropical orchids), and on the optimal light intensity and spectrum required by the different species in each phenological stage, in order to optimize innovative lighting systems (i.e., LEDs).

## Figures and Tables

**Table 1:** Optimal temperatures, suitability for different air conditioning levels in greenhouses (based on minimum night temperature, T_night_), and photoperiodic requirement for flowering in the main cut-flower genera.

	Optimal Temperatures			Greenhouse			Photoperiodic
	T_night_	T_day_	T_substrate_	Cold	Temperate	Heated	Requirement
*Antirrhinum*	8–12	14–18	10–15	X			ND-LDf
*Anthurium*	18–23	23–25	-			X	LDf
*Chrysanthemum*	13–16	20–25	16–18			X	SDo
*Dianthus*	10–12	18–21	10–15	X			ND
*Gerbera*	13–15	20–24	18–20		X		ND-SDf
*Lisianthus*	15–18	24–26	>13			X	LDf
*Matthiola*	10	12–14	-	X			LDf
*Rose*	14–16	20–25	15–18			X	ND
***Geophytes***							
*Alstroemeria*	10–16	18–21	13–16		X		LFf
*Zantedeschia*	10–13	20–25	15–18	X			ND
*Freesia*	10–16	18–24	10–15		X		ND
*Gladiolus*	10–12	16–20	10–15		X		LDf
*Iris*	8–15	15–20	10–13		X		ND
*Lilium*	10–16	18–24	10–15			X	LDf
*Narcissus*	8–15	15–20	10–13				ND
*Ranunculus*	5–10	12–25	<10	X			LDf
*Tulip*	12–18	22–25	8–12	X	X		ND
***Orchids*** ^1^							
*Cattleya*	13–16	16–18	16–18		X		SDo
*Cypripedium*	13–16	16–18	10–14		X		ND
*Cymbidium*	10–14	13–16	10–14	X			ND
*Dendrobium* *Phalaenopsis*	16–18	18–21	16–18			X	SDf, ND

^1^ = dual end use as cut flower and flowering potted plant. Cold greenhouse T_night_ 2–10 °C, Temperate greenhouse T_night_ 10–14 °C, Heated greenhouse T_night_ 16–20 °C. ND = neutral day, SD = short day, LD = long day; o = obligatory, f = facultative.

**Table 2:** Optimal temperatures, suitability for different air conditioning levels in greenhouses (based on minimum night temperature, T_night_), and photoperiodic requirement for flowering in the main flowering potted ornamentals genera.

	Optimal Temperatures			Greenhouse			Photoperiodic
	T_night_	T_day_	T_substrate_	Cold	Temperate	Heated	Requirement
*Rhododendron*	12–14	14–20	15–18	X	X		ND-SD
*Begonia*	15–18	18–21	18–20		X	X	LD
Bromeliaceae	16–20	22–24	18–20			X	ND
*Cyclamen*	12–18	20–22	14–16		X		ND
*Cineraria, Calceolaria*	10–14	15–20	10–15		X		LD
*Euphorbia fulgens*	16–18	20–25	15–20			X	SD
*Gardenia*	14–17	21–23	19–22		X		LD
*Geranium*	14–16	20–25	15–20		X		ND-LD
*Gloxinia*	18–20	20–25	15–20			X	LD
*Hydrangea*	10–18	20–25	18–20	X	X		LD
*Kalanchoe*	14–16	20–25	15–20		X	X	SD
*Poinsettia*	18–20	20–25	18–20			X	SD

Cold greenhouse T_night_ 2–10 °C, Temperate greenhouse T_night_ 10–14 °C, Heated greenhouse T_night_ 16–20 °C. ND = *neutral day*, SD = *short day*, LD = *long day*.

**Table 3:** Optimal substrate temperature, light requirement for seed germination, days required for emergence, and photoperiodic requirement for flowering of the main species of bedding plants.

Scientific Name	Common Name	T Substrate (°C)	Light Requirement	Emergence (Days)	Photoperiodic Requirement
*Ageratum houstonianum*	Floss flower	21–27	L	5–6	LDf
*Amaranthus tricolor*	Edible amaranth	21–24	D/L	8–10	ND
*Antirrhinum majus*	Garden snapdragon	18–21	L	7–14	LDf
*Begonia × semperflorens*	Wax leaf begonia	21–24	L	14–21	ND
*Begonia × tuberhybrida*	Begonia	18	L	15–20	LDo
*Browallia speciosa*	Bush violet	21–24	L	7–10	ND
*Calendula officinalis*	Calendula or Pot marigold	21–27	D	5–10	LDf
*Callistephus chinensis*	China aster	21–27	D/L	8–12	LDf
*Catharanthus roseus*	Periwinkle	21–24	D	14–21	ND
*Celosia cristata*	Cock’s comb	21–27	D/L	8–10	SDo
*Celosia plumosa*	Silver cock’s comb	21–27	D/L	6–10	SDo
*Centaurea cyanus*	Cornflower	18–21	D	10–15	LDf
*Centaurea candidissima*	Velvet centaurea	16–18	D	10–15	LDf
*Cineraria maritima*	Silver ragwort	24	L	10–15	LDf
*Clarkia elegans*	Clarkia or Godetia	18–21	L	5–14	ND
*Cleome spinosa*	Spider flowers	16–29	D/L	7–21	SDf
*Coleus blumei*	Common coleus	18–24	L	10–15	SDo
*Cosmos bipinnatus*	Cosmos	21–24	L	5–14	SDf
*Cynoglossum amabile*	Chinese forget-me-not	16–21	D	5–10	ND
*Dahlia pinnata*	Dahlia	18–24	D/L	5–10	SDf
*Dianthus chinensis*	China pinks	21	D/L	5–7	LDf
*Dimorphotheca aurantiaca*	African daisy	16–21	D	7–15	ND
*Gaillardia pulchella*	Indian blanket	21–27	L	15–20	LDf
*Godetia grandiflora*	Godetia	16	L	14–15	LDf
*Gomphrena globosa*	Globe amaranth	21–27	D	14–20	SDf
*Gypsophila elegans*	Gypsophila	21–27	D/L	10–14	LDo
*Helichrysum bracteatum*	Everlasting flower	21–27	D/L	5–14	ND
*Iberis coronaria*	Iberide annual candytuft	21	D/L	7–14	ND
*Impatiens holstii e I. sultana*	Impatiens	21–24	L	15–18	ND
*Ipomoea purpurea*	Morning-glory	27	D/L	7–14	SDf
*Lathyrus odoratus*	Sweet pea	13–16	D	14–35	LDo
*Lobelia erinus*	Dwarf blue lobelia.	21–24	D/L	14–20	LDo
*Lobularia maritima*	Sweet alyssum	21	D/L	5–14	ND
*Matthiola incana*	Stocks	21	L	7–10	LDf
*Myosotis alpestris*	Alpine forget-me-not	13	D	10–14	ND
*Nicotiana alata*	Flowering tobacco	21	L	7–14	ND-LDf
*Pelargonium hortorum*	Geranium	21–24	L	5–12	ND
*Petunia × hybrida*	Petunia	21–27	L	4–10	LDo-SDf
*Phlox drummondii*	Annual flox	18	D	10–15	LDf
*Portulaca grandiflora*	Portulaca or Rose moss	27	D/L	7–14	ND
*Salpiglossis sinuata*	Salpiglossis /painted tongue	21–24	D	14–21	LDf
*Salvia splendens*	Scarlet sage	21	L	14–21	LDf
*Tagetes erecta*	African marigold	21–27	D/L	5–8	SDf
*Tagetes patula*	French marigold	21–27	D/L	5–8	ND
*Tropaeolum major*	Nasturtium	18	D	10–15	ND
*Verbena × hybrida*	Verbena	18	D	14–20	LDf
*Viola × wittrockiana*	Pansies	18	D	10–20	LDf
*Zinnia elegans*	Zinnia	21–27	D/L	5–10	SDf-LDf

D = dark, L = light; ND = neutral day, SD = short day, LD = long day.

## Data Availability

This review does not report novel data.
